# Draft Genome Sequence of *Streptomyces* sp. Strain Wb2n-11, a Desert Isolate with Broad-Spectrum Antagonism against Soilborne Phytopathogens

**DOI:** 10.1128/genomeA.00860-15

**Published:** 2015-08-06

**Authors:** Martina Köberl, Richard A. White, Sabine Erschen, Tarek F. El-Arabi, Janet K. Jansson, Gabriele Berg

**Affiliations:** a Graz University of Technology, Institute of Environmental Biotechnology, Graz, Austria; b Pacific Northwest National Laboratory, Biological Sciences Division, Richland, Washington, USA; c Ain Shams University, Faculty of Agriculture, Cairo, Egypt; d Heliopolis University, Biotechnology Laboratory, Cairo, Egypt

## Abstract

*Streptomyces* sp. strain Wb2n-11, isolated from native desert soil, exhibited broad-spectrum antagonism against plant pathogenic fungi, bacteria, and nematodes. The 8.2-Mb draft genome reveals genes putatively responsible for its promising biocontrol activity and genes which enable the soil bacterium to directly interact beneficially with plants.

## GENOME ANNOUNCEMENT

*Streptomyces* sp. strain Wb2n-11 was isolated from native desert soil collected in the Sinai desert in Egypt (30°35′01″N; 32°25′49″E) in October 2009, at a depth of 10 to 30 cm. The soil was classified as sand with a clay content of 1.5%, an organic carbon content of <0.2%, and a near-neutral pH of 7.7 (1). Wb2n-11 was selected as broad-spectrum antagonist exhibiting antifungal (*Verticillium dahliae*, *Rhizoctonia solani*, *Fusarium culmorum*), antibacterial (*Ralstonia solanacearum*), and nematicidal (*Meloidogyne incognita*) activity against soilborne phytopathogens (2).

Genomic DNA was extracted using the MasterPure DNA purification kit (Epicentre, Madison, WI, USA) modified with additional cell disruption steps comprising mechanical shredding with glass beads in a FastPrep instrument (MP Biomedicals, Santa Ana, CA, USA) and a lysozyme-based cell wall digestion. PacBio RS libraries with inserts of 8 to 12 kb were constructed and sequenced at GATC Biotech (Konstanz, Germany).

Whole-genome shotgun sequencing yielded 123,881 raw reads with 604,678,994 bp of raw sequence. Assembly was completed with the Hierarchical Genome Assembly Process (HGAP) algorithm implemented in PacBio’s SMRT Analysis software (Pacific Biosciences, Menlo Park, CA, USA) and subsequently upgraded by PBJelly (3). The assembly resulted in five contigs summing to 8,228,099 bp, with a maximum contig size of 7,583,077 bp (*N*_50_ 7,583,077 bp; *N*_90_ 284,642 bp), a 73.5-fold overall coverage, and a G+C content of 71.03%.

The closest relatives based on the full-length 16S rRNA gene sequence are *Streptomyces scopiformis* strain A25 (GenBank accession no. NR_114403) and *Streptomyces enissocaesilis* strain NBRC 100763 (NR_041411), both with 99% sequence similarity. Whole-genome alignment using Mauve (4) revealed conserved regions between Wb2n-11, *Streptomyces griseus* subsp. *griseus* strain NBRC 13350 (NC_010572), and *Streptomyces avermitilis* strain MA-4680 (NC_003155), the closest available reference genomes. However, digital DNA-DNA hybridization using GGDC 2.0 (5–7) against these two genomes excluded the probability of belonging to one of these species.

Annotation was conducted on the RAST Web server using RAST gene calling based on FIGfam version Release70 (8, 9), and additional annotation was completed on the BASys Web server using Glimmer gene prediction (10, 11). The genome annotation contained 7,643 predicted protein-coding genes, 65 tRNA and 21 rRNA loci, and 434 predicted SEED subsystem features.

Wb2n-11 revealed several genes which could contribute to direct and indirect plant growth promotion. We identified genes putatively involved in the biosynthesis of a broad spectrum of antibiotics, such as synthases for bacitracin, tyrocidine, linear and cyclic gramicidin, erythronolide, surfactin, and synthetases for triostin. The genome revealed 14 additional polyketide synthases, some in up to ten copies, and three copies of a dimodular nonribosomal peptide synthase for syntheses of complex secondary metabolites. Wb2n-11 further encodes the production of extracellular cell wall degrading enzymes (chitinase C, extracellular proteases, and glucanases), siderophores, auxin, and spermidine. Wb2n-11 has a complete ectoine biosynthesis and regulation gene cluster that contributes to its survivability under extreme conditions.

### Nucleotide sequence accession numbers.

This whole-genome shotgun project has been deposited in the European Nucleotide Archive under the accession no. CVPB00000000. The version described in this paper is the first version, CVPB01000000.
